# Retraction Note: Weipiling ameliorates gastric precancerous lesions in Atp4a^−/−^ mice

**DOI:** 10.1186/s12906-023-03941-w

**Published:** 2023-04-01

**Authors:** Wei Liu, Zi-ming Zhao, Yuan-liang Liu, Hua-feng Pan, Li-zhu Lin

**Affiliations:** 1 Guangzhou University of Chinese Medicine, Guangzhou, 510405 Guangdong China; 2 Integrative Cancer Centre, the First Affiliated Hospital of Guangzhou, University of Chinese Medicine, Guangzhou, China; 3 Guangdong Province Engineering Technology Research Institute of T.C.M, Guangzhou, 510095 China

**Retraction Note: BMC Complement Med Ther 19, 318 (2019)**

10.1186/s12906-019-2718-y

The Editor has retracted this article because the DAPI, TUNEL and Merged images in Fig. 6 do not appear to correspond with each other. In addition, the authors have not been able to provide original western blotting data that match the images shown in this article. The Editor therefore no longer has confidence in the results and conclusions presented. Zi-ming Zhao and Yuan-liang Liu disagree with this retraction. Wei Liu, Hua-feng Pan and Li-zhu Lin have not responded to correspondence from the Editor about this retraction.

